# Genetic Variation in DEAD-Box Helicase 20 as a Putative Marker of Recurrence in Propensity-Matched Colon Cancer Patients

**DOI:** 10.3390/genes13081404

**Published:** 2022-08-07

**Authors:** Yahya H. Hobani, Amany I. Almars, Walla Alelwani, Eman A. Toraih, Nader A. Nemr, Aly A. M. Shaalan, Manal S. Fawzy, Samy M. Attallah

**Affiliations:** 1 Medical Laboratory Technology, College of Applied Medical Sciences, Jazan University, Jazan 82911, Saudi Arabia; 2 Department of Medical Laboratory Sciences, Faculty of Applied Medical Sciences, King Abdulaziz University, Jeddah 21589, Saudi Arabia; 3 Department of Biochemistry, College of Science, University of Jeddah, Jeddah 80203, Saudi Arabia; 4 Division of Endocrine and Oncologic Surgery, Department of Surgery, Tulane University School of Medicine, New Orleans, LA 70112, USA; 5 Genetics Unit, Department of Histology and Cell Biology, Suez Canal University, Ismailia 41522, Egypt; 6 Endemic and Infectious Diseases Department, Suez Canal University, Ismailia 41522, Egypt; 7 Department of Anatomy, Faculty of Medicine, Jazan University, Jazan 45142, Saudi Arabia; 8 Department of Histology, Faculty of Medicine, Suez Canal University, Ismailia 41522, Egypt; 9 Department of Medical Biochemistry and Molecular Biology, Faculty of Medicine, Suez Canal University, Ismailia 41522, Egypt; 10 Department of Biochemistry, Faculty of Medicine, Northern Border University, Arar 1321, Saudi Arabia; 11 Department of Clinical Pathology, Faculty of Medicine, Mansoura University, Mansoura 35516, Egypt; 12 Clinical Pathology Department, King Fahad Armed Forces Hospital, Jeddah 23311, Saudi Arabia

**Keywords:** colon cancer, *DDX20*, real-time PCR, recurrence, single nucleotide polymorphism, somatic mutation, survival

## Abstract

Variants of the DEAD-Box Helicase 20 (DDX20), one of the microRNAs (miRNAs) machinery genes, can modulate miRNA/target gene expressions and, hence, influence cancer susceptibility and prognosis. Here, we aimed to unravel the association of *DDX20* rs197412 T/C variant with colon cancer risk and/or prognosis in paired samples of 122 colon cancer and non-cancer tissue specimens by TaqMan allelic discrimination analysis. Structural/functional bioinformatic analyses were carried out, followed by a meta-analysis. We found that the T allele was more frequent in cancer tissues compared to control tissues (60.2% vs. 35.7%, *p* < 0.001). Furthermore, the T variant was highly frequent in primary tumors with evidence of recurrence (73% vs. 47.5%, *p* < 0.001). Genetic association models, adjusted by age and sex, revealed that the T allele was associated with a higher risk of developing colon cancer under heterozygote (T/C vs. C/C: OR = 2.35, 95%CI = 1.25–4.44, *p* < 0.001), homozygote (T/T vs. C/C: OR = 7.6, 95%CI = 3.5–16.8, *p* < 0.001), dominant (T/C-T/T vs. C/C: OR = 3.4, 95%CI = 1.87–8.5, *p* < 0.001), and recessive (T/T vs. C/C-T/C: OR = 4.42, 95%CI = 2.29–8.54, *p* = 0.001) models. Kaplan–Meier survival curves showed the shift in the C > T allele to be associated with poor disease-free survival. After adjusting covariates using a multivariate cox regression model, patients harboring C > T somatic mutation were 3.5 times more likely to develop a recurrence (*p* < 0.001). A meta-analysis of nine studies (including ours) showed a higher risk of CRC (81%) in subjects harboring the T/T genotype than in T/C + C/C genotypes, supporting the potential clinical utility of the specified study variant as a biomarker for risk stratification in CRC cases. However, results were not significant in non-colorectal cancers. In conclusion, the *DDX20* rs197412 variant is associated with increased colon cancer risk and a higher likelihood of recurrence in the study population.

## 1. Introduction

The DEAD box proteins are putative RNA helicases characterized by the conserved motif Asp-Glu-Ala-Asp (DEAD) [1]. These motifs mediate ATP-dependent conformational changes associated with RNA unwinding [2]. They are implicated in diverse cellular processes involving an alteration in RNA secondary structure such as translation initiation, nuclear and mitochondrial splicing, and ribosome and spliceosome assembly [3]. Based on their distribution patterns, DEAD box protein family members are believed to be involved in embryogenesis, spermatogenesis, and cellular growth and division [2]. Several RNA helicases have been implicated in the oncogenic process-either through altered expression levels, mutations, or their role in pathways required for tumor initiation, progression, maintenance, or chemosensitivity [4,5,6].

The DEAD-Box Helicase 20 (*DDX20*) gene, located at chromosome 1p13.2, is known as a Gem Nuclear Organelle Associated Protein 3 (*GEMIN3*) and the survival of motor neurons (SMN). *DDX20* gene encodes a 294 amino acid protein that lacks similarity to other known proteins [7]. The DEAD-box protein has an ATPase activity and is a component of the SMN complex. This multiprotein complex is found in the cytoplasm and the nucleus, which are concentrated in bodies called gems. In the cytoplasm, the SMN complex interacts with spliceosomal small nuclear ribonucleoproteins (snRNPs), a critical step for snRNP biogenesis and assembly. Once correctly assembled and modified, the snRNPs recruit the necessary nuclear import receptors and translocate them into the nucleus, where they function in pre-mRNA splicing [7]. While nuclear gems are enigmatic structures of unclear function, they are believed to play a vital role in pre-mRNA splicing, likely by serving in the regeneration or recycling of snRNPs [8].

**Figure 1:**
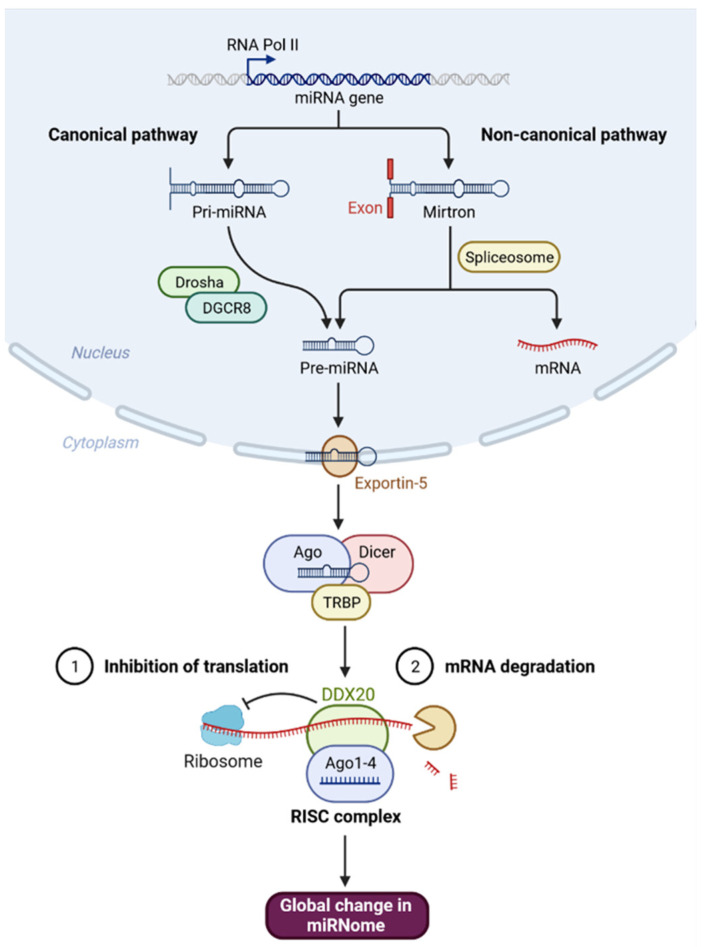
The role of DDX20 in microRNAs biogenesis through forming RISC complex. MicroRNAs (miRNAs) are non-coding RNAs ~22 nucleotides long that bind to target mRNAs, resulting in mRNA degradation or inhibition of mRNA expression. MicroRNAs can be produced from long RNA transcripts. Primary miRNAs (pri-miRNAs), which are 1–2 kb long and contain one or more 70-nt hairpin precursor miRNAs (pre-miRNAs), are excised to pre-miRNAs by DROSHA ribonuclease III (RNase III) and DiGeorge critical region 8 (DGCR8) in the cell nucleus which is exported from the nucleus into the cytoplasm by an exportin-5 (XPO5)/Ran–GTP complex. In the cytoplasm, the endoribonuclease Dicer complex catalyzes these pre-RNAs to form miRNAs. The mature miRNAs are loaded into an argonaute 2 (AGO2) protein, which associates with a TAR RNA-binding protein (TRBP), GEMIN4, and DDX20, and forms an RNA-induced silencing complex (RISC), which plays a crucial role in the repression or degradation of mRNAs. Created with BioRender.com (accessed on 17 April 2022).

Since its discovery, DDX20 has been engaged in all facets of RNA metabolism, from biogenesis to decay [2,4,5,6]. In the biogenesis of miRNAs, DDX20 forms a complex with the Argonaute proteins (Ago1–4) and selectively binds the guide strand of miRNAs to facilitate the formation of miRNA-RNA-induced silencing complex (RISC) [9] (Figure 1). Therefore, single nucleotide polymorphism in miRNA machinery genes can modulate miRNA and target gene expressions and, hence, influence cancer susceptibility, treatment efficacy, and patient prognosis [10]. Aberrant expression and mutations of the *DDX20* gene were associated with cancer development and progression [11,12,13,14]. They exhibited pivotal functions in cellular proliferation and/or neoplastic transformation [2]. Unraveling the role of *DDX20* in cancer may assist in the risk stratification of patients.

Studying the genomic structure of the gene https://www.ncbi.nlm.nih.gov/gene/11218/ (accessed on 10 July 2022) revealed a common missense mutation (rs197412: T/C) that has shown susceptibility to carcinogenesis in previous reports [10,15,16]. This variant was associated with measures of adiposity phenotypes, including body mass index (BMI) [14,17], a well-known risk factor for colorectal cancer [18]. Genetic variants of the *DDX20* gene in tumorigenesis have only been evaluated in a few studies with inconsistent results. Therefore, in the current study, we evaluated the association of the most common genetic polymorphism (rs197412: T/C) in the *DDX20* gene with the risk and prognosis of colon cancer and uncovered its putative regulatory mechanism. The somatic mutation (shifting T allele to C allele or vice versa) during tumorigenesis and recurrence was identified by comparing the alleles frequencies between paired samples. In addition, we performed a meta-analysis to summarize the pooled effect of the SNP in cancer articles.

## 2. Materials and Methods

### 2.1. In Silico Data Analysis of DDX20 Gene and Protein

Genomic localization and structure were analyzed in Ensembl Genomics Browser (https://uswest.ensembl.org/). Transcriptomic isoforms were identified in the Genome Data viewer (www.ncbi.nlm.nih.gov/genome/gdv/browser/). Subcellular localization was predicted in the Compartments database (https://compartments.jensenlab.org/). Protein staining in colon normal and cancer tissues were defined in The Human Protein Atlas (https://www.proteinatlas.org). The 2-D and 3-D structure of the protein was analyzed in the AlphaFold Protein Structure Database (https://alphafold.ebi.ac.uk/entry/Q9UHI6), and protein annotation was visualized in Protter (http://wlab.ethz.ch/protter/start/) (all websites in this paper accessed on 17 April 2022).

To examine the expression range for the *DDX20* gene across all tissues in normal and tumor RNA seq data, TNMplot (https://tnmplot.com/analysis/) for pan-cancer analysis was performed. Prevalence of *DDX20* gene mutation in colorectal carcinoma was identified in cBioPortal for Cancer Genomics (www.cbioportal.org/) using The Cancer Genome Atlas (TCGA), Firehose Legacy dataset for colorectal adenocarcinoma (N = 640 samples). In an attempt to connect mutation status to gene expression changes in solid tumors, the muTarget online tool (www.mutarget.com/analysis/) was used to define top genes which show altered expression in samples harboring a mutated *DDX20* genotype. RNA seq-based gene expression and Mutect2-identified somatic mutation data were obtained from the TCGA repository (https://portal.gdc.cancer.gov/). The Mann–Whitney test was employed.

Overall survival analysis was investigated using a Kaplan–Meier plotter (http://kmplot.com). It is a manually curated database using 165 colorectal cancer patients downloaded from GEO and TCGA. The patient samples are split into two groups according to the auto-select best cutoff option of the online tool, which was at 387 with the expression of DDX20 in RNA-seq experiments ranging from 82 to 1955. In addition, a comparison of survival in patients with gene mutation compared to wild type was also performed. The hazard ratio and 95% confidence intervals, and log-rank *p*-value are calculated. To link gene expression and response to anticancer therapy as a predictive marker for treatment modalities, ROC Plotter (http://www.rocplot.org/) was used (N = 440). Functional enrichment analysis included gene ontology, and pathway analysis was investigated in InnateDB (http://www.innatedb.com/). The protein–protein interaction (PPI) network was analyzed using STRING v11.5 (https://string-db.org/) with an interaction score of a minimum of 0.8.

### 2.2. SNP Selection and Functional Variant Analysis

Extensive mining of the databases of the International HapMap Project (http://www.hapmap.org), and dbSNP (http://www.ncbi.nlm.nih.gov/projects/SNP/) in Ensembl Genomic Database (https://uswest.ensembl.org/) to identify all missense mutations in *DDX20* exons associated with amino acid alterations reported minor allele frequency (MAF) of more than 0.001, and with prior citations. The predicted function was examined by several online tools Polyphen, “Combined Annotation-Dependent Depletion” (CADD), REVEL, metaLR, and mutation assessor (http://www.ensembl.org).

The frequency distributions of *DDX20* genotypes in other populations were investigated in the 1000 Genomes Project (https://www.internationalgenome.org/), a catalog of common human genetic variations. Linkage disequilibrium was examined in GWAS Catalog (www.ebi.ac.uk/gwas/). We used RegulomeDB (https://regulomedb.org/) and HaploReg v4.1 (https://pubs.broadinstitute.org/) to identify variants at each locus that likely influence the regulation of gene expression, incorporating data from the Roadmap Epigenomics (http://www.roadmapepigenomics.org/) and ENCODE (www.encodeproject.org) projects. A 4D Nucleome network and Roadmap ChIP-seq datasets for colonic mucosa were added from http://epigenomegateway.wustl.edu/. Linked SNPs showing the most functional evidence (RegulomeDB score ≤ 4) were then investigated regarding their protein-binding capacity.

### 2.3. Study Subjects and Tissues

A total of 122 Formalin-fixed paraffin-embedded (FFPE) blocks of colon cancer tissue specimens with complete clinicopathological data, archived in the last ten years, were collected from the Suez Canal University hospital pathology lab, Ismailia, and El-laban Pathology Lab, Port-Said, Egypt. Of these, 36/122 (29.5%) patients had positive staining for mutant BRAF protein for *BRAF*^V600E^ mutation. Propensity scores matching analysis yielded two similar datasets of 61 cohorts with primary tumors and 61 matched cohorts with recurrent tumors. Inclusion criteria included the presence of enough tissue samples for a subsequent molecular investigation with complete data. Exclusion criteria were secondary tumors, loss of follow-up, missing the clinicopathological data, samples without paired non-cancer tissues, and samples with insufficient quality of extracted DNA [19]. The demographic data, such as the patient’s age, sex, tumor location, and postoperative course (recurrence and survival), were obtained from the medical records. The International Union Against Cancer TNM staging system [20] was applied for cancer staging. The Declaration of Helsinki’s ethical guidelines were ensured. The local Medical Research Ethics Committee of the Faculty of Medicine, Suez Canal University, approved this study. Patient consent was waived as the included samples in this retrospective study were archived.

### 2.4. Assessment of Time-to-Event Endpoints

Time to progression (TTP) was defined as the interval between the reference date and the date of local, regional or distant relapse/progression, whichever occurs first. Death could be related to primary cancer (primary site or metastatic disease), secondary cancer, protocol treatment, other causes, or death due to an unknown cause. Overall survival (OS) is the time from treatment to death due to any cause. Disease-free survival (DFS) was defined as the time interval between the date of treatment initiation till (a) local, regional, or distant relapse/progression or (b) death due to any cause. Survival times were categorized into short and prolonged; short survival times were defined if ≤48 months after initial treatment.

### 2.5. Propensity Scores Matched the Cohort

Screening of medical records of 1062 samples of colon cancer patients was performed, and demographic and pathological data were abstracted. Propensity-score matching analysis was employed using the MatchIt R package (a one-to-one nearest neighbor algorithm without caliper adjustment). The following covariates were adjusted: age, sex, tumor site, histopathological diagnosis, pathological grade, tumor size, lymph node metastasis, distal metastasis, and BRAF mutation. A propensity-matching score analysis of archived colon specimens yielded two similar datasets (primary and recurrent tumors). The quality of the matches was evaluated by estimating the mean difference and average absolute standardized difference in covariates [19].

### 2.6. DDX20 rs197412 Variant Molecular Analysis

Formalin-fixed paraffin-embedded (FFPE) blocks of tissue specimens were reviewed by a pathologist to distinguish between cancer and non-cancer regions and assess the percentage of tumor cells and normal cells. FFPE sections were cut into a 4-micron thickness in separate Eppendorf tubes (cancer and non-cancer) for molecular analysis. DNA was isolated from these archived FFPE tissue samples using QIAamp DNA FFPE Tissue Kit (Catalog no. 56404, Qiagen, Hilden, Germany). For the 122 patients included in the study, we genotyped 122 cancer and 122 adjacent non-cancer samples. Nearly half of the included patients developed recurrence. The extracted DNA concentration and purity were checked using a “Nanodrop-1000 spectrophotometer (NanoDrop Tech., Wilmington, NC, USA)” and stored at −80 °C for the time of the Real-Time allelic discrimination polymerase chain reaction (PCR) analysis. SNP genotyping was performed in a StepOne™ Real-Time PCR System (Applied Biosystems, Foster City, CA, USA) based on TaqMan assay, following the manufacturer’s recommendations. The specified assay “C____923338_10 (Catalog #: 4351379, Applied Biosystems, Foster City, CA, USA), with specific probe-fluorescence dyes to detect the transition substitution in the context sequence [VIC/FAM]:AATGGTTTTGTGAGAAATAAAGTTA[C/T]TGAACAGAGAGTCCCTGTGTTGGCA according to the build GRCh38. Two independent coauthors blinded to the specimen status of the sample run the PCR. The PCR set was programmed to run the initial denaturation step for 10 min at 95 °C, followed by 40 cycles of amplification for 15 s at 95 °C and annealing for 1 min at 60 °C, and the final step (30 sec) at 60 °C. Internal quality controls and negative controls were applied to ensure genotyping accuracy, and 10% of all samples were randomly selected and genotyped in another run with a 100% concordance rate. SDS software version 1.3.1. (Applied Biosystems, Foster City, CA, USA) was applied for genotyping data analysis. Alleles were compared between paired samples (cancer versus adjacent non-cancer tissues) to identify the genotype alteration (shifting T allele to C allele or vice versa) during the tumorigenesis process and recurrence.

### 2.7. Meta-Analysis

A systematic search in PubMed, GeneCards, NCBI, Varsome, Ensembl, and GWAS was performed. Genotype frequency in cases and controls were abstracted. The pairwise meta-analytical method was carried out using the Mantel–Haenszel method (Random-effects model) and DerSimonian–Laird estimator for tau^2 estimation. Data are presented as relative risk (RR) and 95% confidence interval (CI). Heterogeneity was analyzed using the Cochrane Q test. I^2^ >50% is considered to have significant heterogeneity. A funnel plot and Egger’s test were used to test publication bias. R package “metafor” and “meta” were used.

### 2.8. Statistical Analysis

The McNemar Chi¬-square test was used to compare genotypes in paired cancer and non-cancer adjacent samples of the same patients, while the two-sided Chi-square test/Fisher’s Exact test was used to compare the genotype frequency between recurrent and primary tumor samples. The χ2 test for the Hardy–Weinberg equilibrium (HWE) was applied to test the goodness-of-fit by comparing the observed genotype frequencies with the expected ones among controls. For genetic inheritance model assessment, we performed multivariate logistic regression to compute the odds ratio (OR) and 95% confidence interval (CI), adjusting for age and sex. Kaplan–Meier for SNP genotypes and the log-rank test was used to assess statistically significant differences among survival curves. Hazard ratios with 95% confidence intervals (95% CIs) were estimated using the multivariate Cox proportional hazards model in disease-free survival analysis. All *p*-values reported were two-sided. IBM SPSS Statistics for Windows, Version 27.0. (IBM Corp. Armonk, NY, USA) was applied to conduct the above analyses.

## 3. Results

### 3.1. In Silico Data Analysis

**Figure 2:**
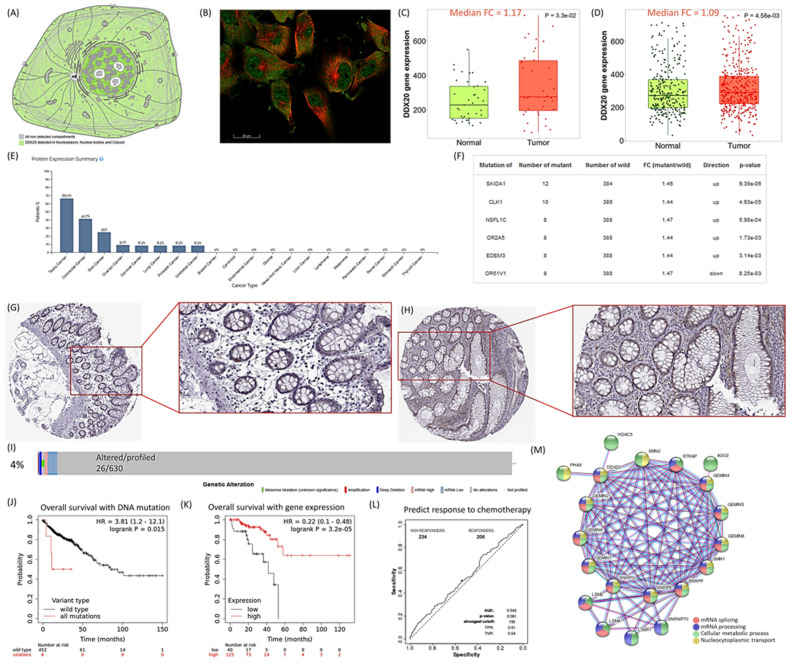
Tissue map of DDX20 in colon tissue. (**A**) Subcellular localization of DDX20 protein. The protein is highly enriched in the cytosol, nucleus (localized in subnuclear structures next to coiled bodies, called Gemini of Cajal bodies (Gems)), and cytoskeleton. (**B**) Immunofluorescent staining of human cell line U-251 MG shows localization to the nucleoplasm, nuclear bodies, and cytosol. DDX20 is stained in green, and microtubules are stained in red. (**C**,**D**) *DDX20* gene expression in colon adenocarcinoma of paired samples (N = 41 paired tumor and 41 adjacent normal tissues) and unpaired samples (N = 469 cancer and 315 normal tissues). (**E**) Expression level in various types of cancers. (**F**) Gene mutations that change the expression of the *DDX20* gene in colon adenocarcinoma (N = 396). (**G**) Immunohistochemistry staining of DDX20 protein in normal colon tissue. Female, 84 years (HPA005516 antibody), normal colon tissue. Negative staining of endothelial cells and peripheral nerves, glandular cells: strong cytoplasmic/membranous staining <25%. (**H**) Immunohistochemistry staining of DDX20 protein in colon cancer. Female, 86 years (HPA005516 antibody), colon adenocarcinoma. Strong cytoplasmic/membranous staining of tumor cells 25–75%. (**I**) Prevalence of *DDX20* gene mutation in colorectal carcinoma was identified in cBioPortal for Cancer Genomics (TCGA, Firehose Legacy, N = 640 samples) (www.cbioportal.org/). (**J**,**K**) Kaplan–Meier plot for overall survival of colorectal adenocarcinoma patients based on DNA mutation of *DDX20* gene (N = 458) and gene expression (N = 165) at the optimum cutoff value (397; expression range of the probe in RNA-seq = 82–1955). The dataset used is in Kaplan–Meier online plotter tool. (**L**) Receiver Operating Characteristics curve analysis for predicting response to anticancer therapy. The analysis included *DDX20* gene expression with any chemotherapy using transcriptomic data of 440 colorectal carcinoma patients. (**M**) Protein–protein interaction network of *DDX20*. Data source: The Human Protein Atlas (https://www.proteinatlas.org/ENSG00000064703-DDX20/subcellular), Kaplan–Meier plotter (http://kmplot.com), ROC Plotter (http://www.rocplot.org/), muTarget (www.mutarget.com/), TNMplot, https://tnmplot.com/analysis/), STRING v11.5 (https://string-db.org/).

The DEAD-box helicase 20 or gem nuclear organelle associated protein 3 gene is located along chromosome 1: 111,754,832–111,775,602 (forward strand). The gene has 21 transcripts, including four protein-coding isoforms (Supplementary Table S1). As depicted in Figure 2, DDX20 protein was localized in the nucleoplasm, nuclear bodies, and cytosol, with intense staining in colon cancer tissues. The *DDX20* gene was significantly overexpressed in colon adenocarcinoma patients compared to controls. In the TCGA colorectal adenocarcinoma, 4% of samples (26 out of 630) were altered. Mutations of *SKIDA1*, *CLK1*, *NSFL1C*, *OR2A5*, *EDEM3*, and *OR51V1* were associated with significant deregulation of *DDX20* gene expression pattern in colon adenocarcinoma patients. Survival analysis showed better survival in wild-type gene variants than mutant versions and in high gene expressor patients. *DDX20* expression was not associated with chemotherapy response with and without radiation therapy.

PPI network analysis revealed that as one of the RNA helicases, DDX20 is involved in multiple aspects of RNA metabolism. It is part of the sm-like protein family complex required to assemble spliceosomal small nuclear ribonucleoproteins. Gene annotations and enriched pathways for DDX20 are shown in Supplementary Table S2. DDX20 plays a crucial role in mRNA splicing via spliceosome, ribonucleoprotein complex assembly, organization, and nucleocytoplasmic transport (Figure 2M).

### 3.2. SNP Selection and Functional Prediction of the Consequence

**Figure 3:**
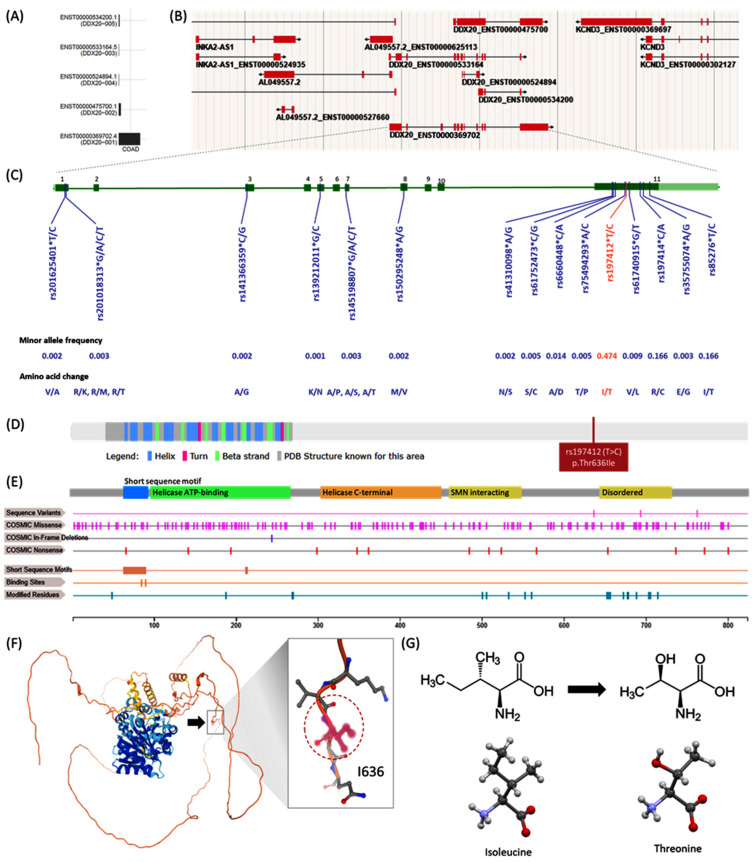
Structural impact of *DDX20* variant. (**A**) Transcript isoforms in colon adenocarcinoma. ENST00000369702.4 is the most expressed transcript. (**B**) Genome browser for active *DDX20* transcripts in a human female 79 years old. (**C**) Missense mutations in *DDX20* with minor allele frequency >0.001. Amino acid alterations are shown. Nearly nine variants were located within exon 11: c.1907T > C (Substitution, position 1907, T→C), which can lead to amino acid mutation p.I636T (Substitution—Missense, position 636, I→T). (**D**) Secondary structure of DDX20 protein of 824 amino acids long and molecular weight of 92,241 Da. The location of the studied SNP rs197412 (p.Thr636Ile) is shown. (**E**) Domains and regions of interest in DDX20 protein (See text). (**F**) 3D structure of DDX20 protein. The amino acid residue 636 (black arrow) is within an unstructured region in isolation. (**G**) Functional prediction of rs197412 missense mutation. Chemical structure of Isoleucine and skeletal formula Structure of L-threonine determined by single crystal X-ray diffraction. Color code: Carbon, C: grey; Hydrogen, H: white; Nitrogen, N: blue; Oxygen, O: red. Model manipulated in Avogadro 1.2 and image generated in CCDC Mercury 3.8. Based on the sequence annotation in the neighborhood (phosphoserine of SER-652, 654, and 656 residues) and physicochemical property of the reference and variant residues and reside alteration implicated, the change from medium size and hydrophobic Isoleucine to medium size and polar Threonine was forecasted to be likely benign by functional predicted website tools. Data source: UniProt Q9UHI6 (www.uniprot.org), COSMIC-3D (cancer.sanger.ac.uk/cosmic3d/), AlphaFold Protein Structure Database (https://alphafold.ebi.ac.uk/entry/Q9UHI6), and Wikipedia (https://en.wikipedia.org/wiki/).

Through database mining, we identified 563 missense mutations in *DDX20* exons associated with amino acid alterations (Supplementary Table S3). Of these, 15 SNPs have a reported MAF of more than 0.001 and only 4 SNPs have MAF over 0.01; namely rs197412 (T/C; Ile636Thr; MAF = 0.474), rs85276 (T/C; Ile762Thr; MAF = 0.166), rs197414 (C/A; Arg693Ser; MAF = 0.166), and rs6660448 (C/A; Ala592Asp; MAF = 0.014). All were predicted to be benign and tolerated mutation by Polyphen, CADD, REVEL, metaLR, and mutation assessor. Two variants, rs197412 and rs197414, had prior citations. Since rs197412 SNP was associated with some cancers and obesity with the highest MAF, it was selected to be investigated in our colorectal cancer patients. Secondary and tertiary structures of DDX20 protein are demonstrated in Figure 3. ENST00000369702 was the most expressed transcript in colon adenocarcinoma.

Regarding the significant domains and regions of interest in DDX20 protein, the DEAD-box helicase domain (aa 93–264) is involved in various aspects of RNA metabolism, including nuclear transcription, pre mRNA splicing, ribosome biogenesis, nucleocytoplasmic transport, translation, RNA decay, and organellar gene expression. Helicase conserved C-terminal domain (aa 299–448) is an integral part of the helicase. The SMN complex (aa 456–548) plays a catalyst in assembling small nuclear ribonucleoproteins (snRNPs), the building blocks of the spliceosome. Thereby, DDX20 plays an essential role in the splicing of cellular pre-mRNAs. As the result of posttranslational modifications, phosphothreonine is produced at THR-552, 688, and 705, in addition to phosphorylation of 16 serine residues at SER-48, 187, 268, 269, 500, 505, 532, 560, 652, 654, 656, 672, 677, 678, 703, and 714. There are two short sequence motifs: Q motif (aa 62–90) and DEAD box (211–214). Q motif is a conserved cluster of nine amino acids with invariant glutamine located N-terminally of motif I. An additional highly conserved but isolated aromatic residue is also found upstream of these nine residues. The Q motif control ATP binding and hydrolysis, and, therefore, it represents a potential mechanism for regulating helicase activity. The D-E-A-D motif is involved in the ATP binding and ATPase activity and the interaction with nucleic acids (Figure 3E).

The studied rs197412 missense variant is located in the coding sequence of exon 11 at chromosome 1 position 111766331. This SNP covers the following transcript isoforms ENST00000369702.5, ENST00000679724.1, and ENST00000680627.1 of the *DDX20* gene. It results from substituting T with C (c.1907T > C). The ATT/ACT codon change leads to an amino acid change from isoleucine to threonine at position 636 (p.Ile636Thr) of DDX20 protein. Isoleucine is non-polar, uncharged (at physiological pH), branched chain, and aliphatic amino acid. In contrast, threonine is a polar, uncharged amino acid. The threonine residue is susceptible to numerous post-transcriptional modifications such as phosphorylation and O-linked glycosylation. Recent investigations have shown that substituting Threonine for Isoleucine in other proteins may affect a part of the macromolecular substrate binding site. Because Threonine has a hydroxyl group in its side chain, it is possible that this hydroxyl group makes new hydrogen bonds and disturbs the substrate-binding site (Figure 3F,G).

**Figure 4:**
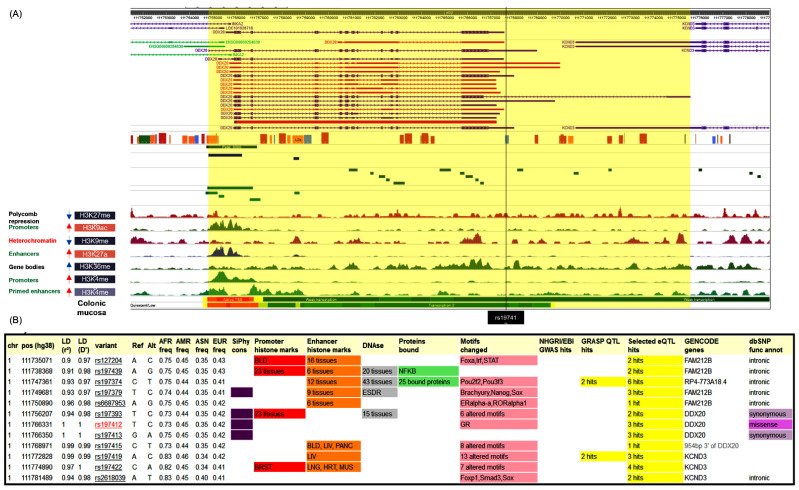
Predicted functional impact of rs197412 on chromatin status and regulatory motifs. (**A**) Tissue-specific epigenomic enrichment. ChIP-seq signal tracks at poised and active promoters near the *DDX20* gene are shown. (**B**) The SNP was in linkage disequilibrium with 11 genetic variants (r2 > 0.8). rs197412 SNP was shown to cause regulatory motif alteration of the glucocorticoid receptor (GR). Data source: Roadmap Epigenomics (http://www.roadmapepigenomics.org/) and ENCODE (www.encodeproject.org) projects, RegulomeDB (https://regulomedb.org/), HaploReg v4.1 (https://pubs.broadinstitute.org/) (all online tools were accessed on 17 April 2020).

Next, we examined 31 major epigenetic histone modifications in colonic mucosa (methylation or acetylation) involved in gene regulation. They are presented as peak density. Across the *DDX20* gene, seven transposon-linked histone marks evolved coordinated depending on their functional roles. Active chromatin peaks for H3K9ac, H3K27ac, and H3K4me3 were enriched at TSS-proximal regions (promoters and enhancers). H3K4me1 was enriched at active and primed enhancers. In contrast, H3K26me3 and H3K9me3 were tagged at heterochromatin sites. In colon mucosa and smooth muscle, the rs197412 does not influence the activity of promoters or enhancers, unlike other organs and cell types such as the lungs, heart, liver, and the brain, where the SNP overlaps with an active promoter and enhancer histone markers (H3K27ac and H3K9ac). However, rs197412 SNP was shown to cause regulatory motif alteration in the glucocorticoid receptor (GR) (Figure 4). No data were available for the expression quantitative trait loci (eQTLs) analysis associated with the studied SNP.

**Figure 5:**
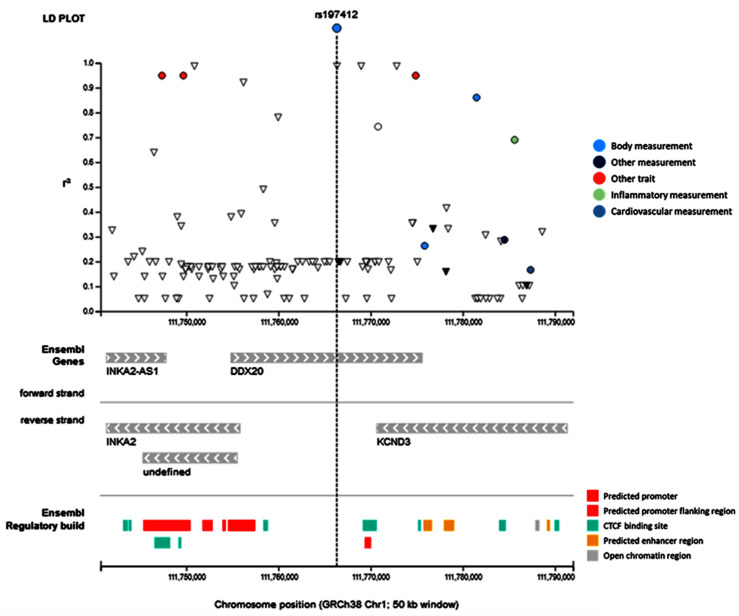
Linkage disequilibrium analysis. *DDX20* rs197412 SNP was in linkage disequilibrium with other SNPs associated with multiple phenotypes such as educational attainment, medication use of opioids, multisite chronic pain, body mass index, and adult body size.

Linkage disequilibrium analysis in the GWAS catalog revealed its association with other nearby variants. None of them were cited in colorectal cancer patients (Figure 5).

### 3.3. Characteristics of Propensity-Matched Samples

The study included paired samples of 122 colon cancer and 122 paired control tissue specimens. Their mean age was 56.8 years ± 12.1, and 67.2% were males. Of these 122 patients, 26.2% died during the follow-up period of over 5.5 years duration. Baseline characteristics of matched cohorts are demonstrated in Table 1.

### 3.4. DDX20 rs197412T/C: A Diagnostic and Prognostic Biomarker

**Figure 6:**
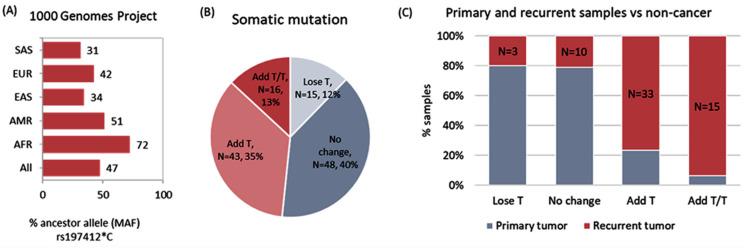
Mutation screening of *DDX20* rs197412(T/C) variant. (**A**) Minor allele frequency (MAF) in different populations. AFR: African, AMR: American, EAS: East Asian, EUR: European, SAS: South Asian. Data source: 1000 Genomes Project (https://www.internationalgenome.org/). (**B**) Frequency of somatic mutation shift of genotype from C to T in tumor samples. Cancer samples were compared to paired non-cancer samples. (**C**) Comparison between the rate of mutation shift from C to T in recurrent (N = 61) and primary (N = 61) tumor specimens.

We identified no evidence of departure from Hardy–Weinberg equilibrium (*p* = 0.2). MAF (C allele) accounted for 0.52 in the study population. Based on the 1000 Genome Project, the *DDX20* rs197412T/C was 0.72 in Africans, 0.34 in East Asians, 0.31 in South Asian, 0.51 in Americans, and 0.42 in Europeans (Figure 6A). On pairwise comparison between cancer and non-cancer adjacent colon tissues, the T allele was more frequent in cancer tissues compared to control tissues (60.2% vs. 35.7%, *p* < 0.001). Correspondingly, T/T genotype was more prevalent in cancer specimens (37.7% vs. 12.3%, *p* < 0.001) (Table 2), thus highlighting the role of the SNP as a diagnostic biomarker. Furthermore, the T variant was highly frequent in cancer samples with a history of recurrence (73% vs. 47.5%, *p* < 0.001) (Table 2). Therefore, these results demonstrated the putative role of *DDX20* rs197412T/C as a prognostic marker, with homozygote patients carrying the T allele having a higher risk of recurrence.

### 3.5. Impact of Genotypes on Cancer Risk

Analysis of various genetic association models, adjusted by age and sex, revealed that the T variant was associated with higher susceptibility to developing colon cancer under heterozygote comparison (T/C vs. C/C: OR = 2.35, 95%CI = 1.25–4.44, *p* < 0.001), homozygote comparison (T/T vs. C/C: OR = 7.6, 95%CI = 3.5–16.8, *p* < 0.001), dominant model (T/C-T/T vs. C/C: OR = 3.4, 95%CI = 1.87–8.5, *p* < 0.001), and recessive model (T/T vs. C/C-T/C: OR = 4.42, 95%CI = 2.29–8.54, *p* = 0.001).

### 3.6. Somatic Mutation Burden Analysis

Nearly half of the tumor samples (N = 59, 48.3%) exhibited the addition of at least one T allele in the tumor specimen; 43 patients (35.2%) had C to T switch, and 16 (13.1%) samples had C/C to T/T switch in cancer samples compared to paired normal tissue (Figure 6B). This somatic mutation was more likely to occur in recurrent samples than in primary tumor tissues (Figure 6C).

### 3.7. DDX20 Variant Is a Poor Prognostic Marker

**Figure 7:**
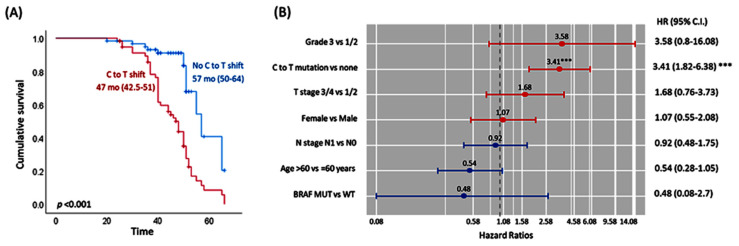
Survival analysis of colon cancer patients. (**A**) Kaplan–Meier survival curves illustrating the differential effect of *DDX20* somatic mutation on disease-free survival. Survival times are shown as median and interquartile ranges. Log Rank test was used to compare the difference between the groups. (**B**) Independent predictor risk factors for disease-free survival. Cox Proportional Hazard Regression analysis was performed. Hazard ratio and confidence interval are reported. *** indicates *p* < 0.001.

Somatic mutation of *DDX20* in cancer tissues was significantly associated with recurrence. As depicted in Figure 7A, Kaplan–Meier survival curves showed the shift in the C to T allele associated with poor disease-free survival (median: 47 months vs. 57 months, *p* <0.001). After adjustment of covariates using the multivariate cox regression model, patients harboring the C > T shift were 3.5 times more likely to develop a recurrence (*p* < 0.001) (Figure 7B).

### 3.8. Meta-Analysis Results

**Figure 8:**
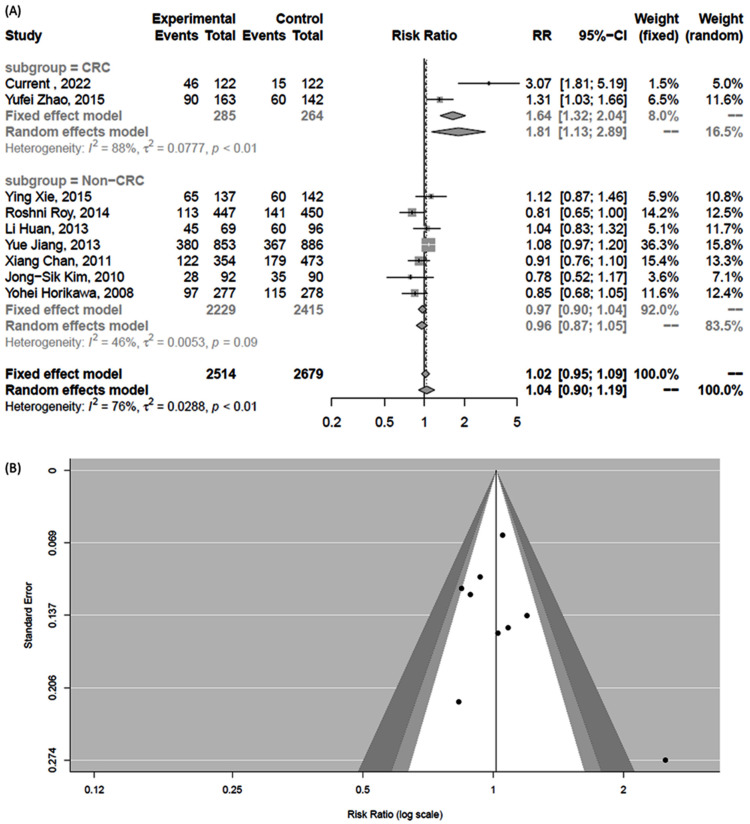
Pooled pairwise comparison for *DDX20* rs197412 polymorphism in cancer versus non-cancer tissues. (**A**) Random forest for pooled effect size. Relative risk and 95% confidence interval were estimated. Data was stratified based on the type of cancer: colorectal versus non-colorectal cancer [10,21,22,23,24,25,26]. (**B**) Funnel plot to test for publication bias.

A systematic search for *DDX20* rs197412 yielded six colorectal cancer and 12 non-cancer articles. Of these, eight depicted genotype counts. Nine studies were analyzed (including ours) [10,21,22,23,24,25,26]. It is worth noting that there were two articles in Chinese (Xiang Chan and colleagues worked on breast cancer, and Li Huan et al. worked on the lymphoma); the authors could not reach the original articles, but the required genotyping data were retrieved from Zhu et al. [23]. Pairwise comparison between cancer and non-cancer patients showed a higher risk of colorectal cancer in subjects harboring the T/T genotype than T/C + C/C genotypes (RR = 1.81, 95%CI = 1.13–2.89, *p* <0.01). However, results were not significant in non-colorectal cancers (RR = 0.96, 95%CI = 0.87–1.05). The funnel plot and Egger’s test did not show publication bias (Figure 8).

## 4. Discussion

Colorectal cancer ranks as the second leading cause of cancer-related mortality in western countries and the third most common malignancy globally [27]. Identifying predictive markers for cancer recurrence is crucial to inform future strategies in controlling the disease’s burden and discovering novel therapeutic plans. According to the current oncological standards, colorectal cancer prognosis depends on the time of diagnosis, as early colorectal cancer screening is related to reduced mortality [28]. Therefore, there is a need to find new reliable biomarkers which can predict risks of early recurrence and metastasis in colorectal cancer patients, biomarkers that could be easily incorporated into the routine diagnostic workup. In the current study, we investigated the potential association of *DDX20* rs197412 polymorphism with colon cancer susceptibility and progression. We found that T/T genotype was associated with a higher risk of tumor recurrence.

The 824-aa DEAD-box ribonucleoprotein, DDX20, is involved in the processing of miRNA precursors through their interaction with the key components of the RNA-induced silencing complex [26]. Genetic variations in *DDX20* can potentially disrupt the fine-tune regulation of multiple biological cellular processes, leading to cancer development and susceptibility [15]. The rs197412 located in exon 11 of *the DDX20* gene induces Isoleucine to Threonine substitution at 636 amino acid position through the T to C transition. The rs197412 SNP of *DDX20* has been identified for its association with increased cancer susceptibility and outcomes [26,29,30]. In colorectal cancer studies, the TT genotype carriers of rs197412 located in *DDX20* exhibited a significant 69% increased risk of CRC compared with CT + CC carrier [10]. In addition, CRC patients harboring the T allele had a 47% higher chance of recurrence [31]. In contrast, *DDX20* gene variants rs197412 T/C (Thr636Ile), rs197414 C/A (p.Ser693Arg), and rs197388 (c.12 + 841T > A) were not associated with overall and progression-free survivals in colorectal patients of Canadian [32] or Korean descent [33]. The rs197412 polymorphism was not associated with outcomes of CRC patients receiving 5-fluorouracil (5-FU) and irinotecan-based chemotherapy [31,34].

In other types of cancer, the SNP (rs197412) was found to be associated with susceptibility to renal [26] and esophageal cancers [30]. Likewise, Roy et al. reported that the rs197412*C/C variant confers a 40% reduced risk of oral cancer [22]. Individuals with *DDX20* non-synonymous rs197412 variant genotypes exhibited 42% reduced oral premalignant risk (OR, 0.58; 95% CI, 0.33–0.99) compared to individuals with wild type genotype (T/T) [15]. *DDX20* rs197412 T variant was also found consistently associated with overall survival in patients with RCC [29] and non-Hodgkin’s lymphoma (NHL) [16], recurrence-free survival in bladder cancer [35], and both overall survival and progression-free survival in patients with esophageal adenocarcinoma [30]. However, other studies yielded inconclusive results. Significant association with cancer susceptibility was not detected in patients with hepatocellular carcinoma [36] or esophageal cancer [12,23].

Additionally, pooled results of a prior meta-analysis showed no association of rs197412 polymorphism with cancer risk [23]. In the current study, we performed a meta-analysis to investigate the role of the SNP across different types of cancer. The T/T genotype was significantly associated with an 81% increased risk of colorectal cancer, while the pooled result was borderline for the non-colorectal cancers, highlighting the possibility of differential cell type-specific behavior. Further genotype-to-phenotype analyses are required to characterize better tissue-specific changes caused by this polymorphism and possibly other linked polymorphisms on its gene product.

Our in silico data analysis showed that the DDX20 is a multifunctional protein that interacts with Epstein–Barr virus nuclear proteins (EBNA2/EBNA3) and is part of the spliceosomal small nuclear ribonucleoproteins complex. As a nuclear receptor co-regulator, DDX20 interacts with the nuclear receptor steroidogenic factor-1 (SF-1, NR5A1), a key regulator of reproductive development, and represses its transcriptional activity. DDX20 plays a vital role in nucleocytoplasmic transportation, negative regulation of cell proliferation and transcription by RNA polymerase II, positive regulation of apoptotic process, steroid biosynthetic process, RNA processing, and spliceosomal snRNP assembly. The mechanism by which this SNP modifies the CRC risk remains unclear. The rs197412 is located in exon 11, a hot spot for multiple missense mutations. Based on our analysis using the PolyPhen-2 tool and others, this non-synonymous polymorphism is not predicted to affect the protein function. However, this substitution, located in the C-terminal domain of the protein, might change mRNA stability and expression or protein function. The deregulated gene expression could alter global miRNA homeostasis and have a significant effect on cellular signaling pathways, thereby modifying CRC carcinogenesis. Despite several articles reporting associations, none of the SNP studies in miRNA processing machinery have been independently validated, nor have the biological mechanisms of how they affect miRNA maturation and carcinogenesis. Therefore, functional studies for miRNA biogenesis genes, such as *DDX20*, are warranted. Another putative mechanism is that the rs197412 was in linkage disequilibrium with other nearby SNPs, e.g., rs197414, which was associated with a higher risk of bladder cancer [37] and esophageal cancer [12]. Further experiments of tagged SNPs and haplotype blocks would unravel gene–gene interactions.

It is worth noting that colon cancer is a complex disorder with a multistage process that involves multiple genes/pathways and variants. The “candidate gene approach” that considers one gene/variant at a time may not be able to identify the modest impact associated with each variant. In this sense, taking a multigenic approach in “pathway-based association studies” to identify signatures of genetic variations as predictors of cancer risk is essential. Furthermore, environmental exposures could not be determined in this study; future studies are required to unravel the gene-environment interaction to clarify the big picture.

## 5. Conclusions

In the present study, *DDX20* rs197412 was an independent prognostic marker for colon cancer recurrence-free survival. This information may help identify new biological pathways influencing colon cancer outcomes. However, studies assessing the functional effect of the *DDX20* rs197412 are needed, and further experiments in larger cohorts with various ethnic groups are required to confirm our conclusions due to the presence of contradictory studies in the literature.

## Figures and Tables

**Table 1:** Baseline characteristics of the study population in primary and recurrent samples.

Variable		Total (N = 122)	Primary (N = 61)	Recurrent (N = 61)	*p*-Value
Age (y)	≤60	77 (63.1)	41 (67.2)	36 (59)	0.45
	>60	45 (36.9)	20 (32.8)	25 (41)	
Sex	Male	82 (67.2)	42 (68.9)	40 (65.6)	0.85
	Female	40 (32.8)	19 (31.1)	21 (34.4)	
Location	Right	67 (54.9)	35 (57.4)	32 (52.5)	0.49
	Transverse	7 (5.7)	2 (3.3)	5 (8.2)	
	Left	48 (39.3)	24 (39.3)	24 (39.3)	
Type	Adenocarcinoma	84 (68.9)	41 (67.2)	43 (70.5)	0.75
	Mucinous	16 (13.1)	7 (11.5)	9 (14.8)	
	Signet cell	18 (14.8)	11 (18)	7 (11.5)	
	Undifferentiated	4 (3.3)	2 (3.3)	2 (3.3)	
Grade	G1/2	83 (68)	41 (67.2)	42 (68.9)	0.84
	G3	39 (32)	20 (32.8)	19 (31.1)	
T stage	T1/2	65 (53.3)	32 (52.5)	33 (54.1)	0.85
	T3/4	57 (46.7)	29 (47.5)	28 (45.9)	
N stage	N0	45 (36.9)	20 (32.8)	25 (41)	0.45
	N1	77 (63.1)	41 (67.2)	36 (59)	
M stage	M0	98 (80.3)	49 (80.3)	49 (80.3)	0.82
	M1	24 (19.7)	12 (19.7)	12 (19.7)	
Lymphovascular invasion	No	82 (67.2)	41 (67.2)	41 (67.2)	0.85
	Yes	40 (32.8)	20 (32.8)	20 (32.8)	
Duke’s stage	A/B	63 (51.6)	31 (50.8)	32 (52.5)	0.85
	C/D	59 (48.4)	30 (49.2)	29 (47.5)	
BRAF mutation	Wild type	86 (70.5)	42 (68.9)	44 (72.1)	0.84
	Mutant	36 (29.5)	19 (31.1)	17 (27.9)	
Relapse	No	83 (68)	44 (72.1)	39 (63.9)	0.43
	Yes	39 (32)	17 (27.9)	22 (36.1)	
Mortality	Survived	90 (73.8)	49 (80.3)	41 (67.2)	0.15
	Died	32 (26.2)	12 (19.7)	20 (32.8)	
Disease-free survival	Prolonged > 2y	46 (39)	21 (36.2)	25 (41.7)	0.57
	Short ≤ 2y	72 (61)	37 (63.8)	35 (58.3)	
Overall survival	Prolonged > 2y	61 (52.6)	30 (51.7)	31 (53.4)	0.85
	Short ≤ 2y	55 (47.4)	28 (48.3)	27 (46.6)	

Data are presented as frequency (percentage). A two sided-Chi-square test was used. Statistical significance was set at a *p*-value below 0.05.

**Table 2:** Genotype and allele frequencies of *DDX20* rs197412T/C.

Frequencies	Total (N = 244)	Control Samples (N = 122)	Tumor Samples (N = 122)	*p*-Value	Primary (N = 61)	Recurrent (N = 61)	*p*-Value
Alleles							
C	254 (52)	157 (64.3)	97 (39.8)	**<0.001**	64 (52.5)	33 (27)	**<0.001**
T	234 (48)	87 (35.7)	147 (60.2)		58 (47.5)	89 (73)	
Genotypes							
C/C	71 (29.1)	50 (41.0)	21 (17.2)	**<0.001**	17 (27.9)	4 (6.6)	**<0.001**
C/T	112 (45.9)	57 (46.7)	55 (45.1)		30 (49.2)	25 (41)	
T/T	61 (25.0)	15 (12.3)	46 (37.7)		14 (23)	32 (52.5)	

Data are presented as frequency (percentage). The McNemar-Bowker and Two-sided Chi-square tests were used. The bold values indicate statistical significance at *p*-values below 0.05.

## Data Availability

All generated data in this study are included in the article and Supplementary Materials.

## Supplementary Materials

The following supporting information can be downloaded at: https://www.mdpi.com/article/10.3390/genes13081404/s1, Table S1: Transcript isoforms of *DDX20* gene; Table S2: Gene ontology and pathway analysis of *DDX20* gene; Table S3: Missense single nucleotide polymorphisms in *DDX20* gene.
