# Supplementary material for: Genetic Variation in DEAD-Box Helicase 20 as a Putative Marker of Recurrence in Propensity-Matched Colon Cancer Patients
Source: Genes (Basel). 2022 Aug 7;13(8):1404. doi: 10.3390/genes13081404 (PMC9407271; doi:10.3390/genes13081404)
Supplement: Supplementary file 1 [file genes-13-01404-s001.zip › Suppl. Tables S1 and S2.pdf]

Supplementary Table S1. Transcript isoforms of *DDX20* gene

Transcript ID	Name	bp	Protein	Biotype
ENST00000369702.5	**DDX20-201**	3600	824 aa	Protein coding
ENST00000679724.1	DDX20-209	4658	824 aa	Protein coding
ENST00000680627.1	DDX20-216	3322	754 aa	Protein coding
ENST00000680518.1	DDX20-215	2817	423 aa	Protein coding
ENST00000681559.1	DDX20-220	5375	279 aa	Nonsense mediated decay
ENST00000533164.6	**DDX20-204**	3226	138 aa	Nonsense mediated decay
ENST00000679576.1	DDX20-208	3153	193 aa	Nonsense mediated decay
ENST00000680936.1	DDX20-217	3076	294 aa	Nonsense mediated decay
ENST00000680383.1	DDX20-213	2755	345 aa	Nonsense mediated decay
ENST00000679498.1	DDX20-207	7416	No protein	Retained intron
ENST00000679774.1	DDX20-210	7183	No protein	Retained intron
ENST00000534200.2	**DDX20-205**	4697	No protein	Retained intron
ENST00000475700.1	**DDX20-202**	4332	No protein	Retained intron
ENST00000680415.1	DDX20-214	4322	No protein	Retained intron
ENST00000681747.1	DDX20-221	3818	No protein	Retained intron
ENST00000680038.1	DDX20-211	3810	No protein	Retained intron
ENST00000681529.1	DDX20-219	3522	No protein	Retained intron
ENST00000680317.1	DDX20-212	3486	No protein	Retained intron
ENST00000679381.1	DDX20-206	3422	No protein	Retained intron
ENST00000680983.1	DDX20-218	2688	No protein	Retained intron
ENST00000524894.2	**DDX20-203**	894	No protein	Retained intron

Data source: Ensemble Genome Browser (www.ensembl.org). The bold isoforms are transcribed in colon adenocarcinoma.

Table S2. Gene ontology and pathway analysis of *DDX20* gene

GO domain	Accession	GO term
Molecular Function	GO:0003676	Nucleic acid-binding
	GO:0003677	DNA binding
	GO:0004004	ATP-dependent RNA helicase activity
	GO:0004386	Helicase activity
	GO:0005515	Protein binding
	GO:0005524	ATP binding
	GO:0019904	Protein domain-specific binding
	GO:0042826	Histone deacetylase binding
Biological Process	GO:0000122	Negative regulation of transcription from RNA polymerase II promoter
	GO:0000244	Spliceosomal tri-snRNP complex assembly
	GO:0000387	Spliceosomal snRNP assembly
	GO:0006200	ATP catabolic process
	GO:0006396	RNA processing
	GO:0008152	Metabolic process
	GO:0010467	Gene expression
	GO:0016070	RNA metabolic process
	GO:0034660	ncRNA metabolic process
	GO:0043065	Positive regulation of the apoptotic process
	GO:0045892	Negative regulation of transcription, DNA-templated
	GO:0048477	Oogenesis
	GO:0050810	Regulation of steroid biosynthetic process
Cellular Component	GO:0005634	Nucleus
	GO:0005654	Nucleoplasm
	GO:0005737	Cytoplasm
	GO:0005829	Cytosol
	GO:0005856	Cytoskeleton
	GO:0016020	Membrane
	GO:0017053	Transcriptional repressor complex
	GO:0032797	SMN complex
	GO:0034719	SMN-Sm protein complex
	GO:0097504	Gemini of coiled bodies
REACTOME	REACT_11066	snRNP Assembly pathway
	REACT_11052	Metabolism of non-coding RNA pathway
	REACT_71	Gene Expression pathway
KEGG	hsa03013	RNA transport pathway

Data source: InnateDB (http://www.innatedb.com/).
